# OsSpo11-4, a Rice Homologue of the Archaeal TopVIA Protein, Mediates Double-Strand DNA Cleavage and Interacts with OsTopVIB

**DOI:** 10.1371/journal.pone.0020327

**Published:** 2011-05-26

**Authors:** Xiao Jing An, Zhu Yun Deng, Tai Wang

**Affiliations:** 1 Research Center of Molecular and Developmental Biology, Key Laboratory of Photosynthesis and Environmental Molecular Physiology, Institute of Botany, Chinese Academy of Sciences, Beijing, China; 2 Graduate School of Chinese Academy of Sciences, Beijing, China; University of Texas-Houston Medical School, United States of America

## Abstract

DNA topoisomerase VI from Archaea, a heterotetrameric complex composed of two TopVIA and two TopVIB subunits, is involved in altering DNA topology during replication, transcription and chromosome segregation by catalyzing DNA strand transfer through transient double-strand breaks. The sequenced yeast and animal genomes encode only one homologue of the archaeal TopVIA subunit, namely Spo11, and no homologue of the archaeal TopVIB subunit. In yeast, Spo11 is essential for initiating meiotic recombination and this function appears conserved among other eukaryotes. In contrast to yeast and animals, studies in *Arabidopsis* and rice have identified three Spo11/TopVIA homologues and one TopVIB homologue in plants. Here, we further identified two novel Spo11/TopVIA homologues (named OsSpo11-4 and OsSpo11-5, respectively) that exist just in the monocot model plant *Oryza sativa*, indicating that at least five Spo11/TopVIA homologues are present in the rice genome. To reveal the biochemical function of the two novel Spo11/TopVIA homologues, we first examined the interactions among OsSpo11-1, OsSpo11-4, OsSpo11-5, and OsTopVIB by yeast two-hybrid assay. The results showed that OsSpo11-4 and OsTopVIB can self-interact strongly and among the 3 examined OsSpo11 proteins, only OsSpo11-4 interacted with OsTopVIB. Pull-down assay confirmed the interaction between OsSpo11-4 and OsTopVIB, which indicates that OsSpo11-4 may interact with OsTopVIB *in vivo*. Further *in vitro* enzymatic analysis revealed that among the above 4 proteins, only OsSpo11-4 exhibited double-strand DNA cleavage activity and its enzymatic activity appears dependent on Mg^2+^ and independent of OsTopVIB, despite its interaction with OsTopVIB. We further analyzed the biological function of OsSpo11-4 by RNA interference and found that down-regulated expression of OsSpo11-4 led to defects in male meiosis, indicating OsSpo11-4 is required for meiosis.

## Introduction

Topoisomerase VI (TopVI), originally identified from the hyperthermophilic archaeon *Sulfolobus shibatae*, regulates DNA topology by catalyzing DNA strand transfer through transient double-strand breaks in the presence of Mg^2+^ and ATP and is the only topoisomerase that can relax positively supercoiled DNA, thereby being essential to DNA replication, transcription and chromosome segregation [1], [2]. TopVI consists of 2 distinct subunits, TopVIA and TopVIB. TopVIA is a catalytic subunit responsible for DNA binding and cleavage, and TopVIB is involved in ATP binding and hydrolysis. TopVIA is characterized by 5 conserved functional motifs, I to V, with 2 domains: CAP (catabolite activator protein) and toprim (topoisomerases as well as DNA primases). Motif I and II constitute a helix-turn-helix fold of the CAP domain, which exists widely in DNA-binding proteins. The conserved toprim domain spanning motifs III to V is involved in DNA binding and cleavage, with the DXD sequence responsible for coordinating metal ions [3], [4], [5]. The N-terminal region of *S. shibatae* TopVIB is characterized by a Bergerat fold, which is found in proteins of the GHKL family (DNA gyrase, Hsp90, bacterial histidine kinases and MutL families) [6]. The Bergerat fold, consisting of 3 motifs (B1 to B3), is responsible for ATP binding and hydrolysis [6], [7]. The C-terminus of TopVIB is structurally homologous to that of GyrB, termed the transducer domain (motif B4), which is required for the transmission of structural signals and conformational changes [8].

The eukaryotic homologue of archaeal TopVIA is Spo11, which was first identified in *Saccharomyces cerevisiae* and can induce meiotic DNA double strand breaks (DSBs) by a topoisomerase II-like mechanism [9]. Spo11 functions as a dimer to cleave DNA double strands and each molecule remains covalently attached to one DSB end after generation of the break [10]. The Spo11-induced DSBs are required for initiation of homologous recombination and thus necessary for subsequent chromosome segregation in yeast meiosis [9], [11], [12]. Spo11 homologues have been identified in diverse eukaryotes, including *Schizosaccharomyces pombe* [2], [13], [14], *Caenorhabditis elegans* [15], *Homo sapiens* [16], [17], *Mus musculus* [18], *Drosophila melanogaster* [19], *Coprinus cinereus* [20], *Arabidopsis thaliana* [21], [22], *Oryza sativa* [23] and a few protists [24]. Studies in *C. cenereus*, *D. melanogaster*, *C. elegans* and *A. thaliana* also demonstrated that mutation in the *SPO11* genes causes defects in meiotic recombination [19], [20], [25], [26], suggesting that the function of Spo11 in initiating meiotic recombination is likely universally conserved among eukaryotes.

Analyses of eukaryotic genomes revealed the presence of at least 3 TopVIA/Spo11 homologues and 1 TopVIB homologue in higher plants, as opposed to 1 single TopVIA/Spo11 homologue and no TopVIB homologue in fungi and animals [27]. In *Arabidopsis*, 3 TopVIA/Spo11 homologues (AtSpo11-1, AtSpo11-2, and AtSpo11-3) and 1 TopVIB homologue (AtTopVIB) have been identified [21], [22]. AtSpo11-1 and AtSpo11-2 are required for meiotic recombination and accurate chromosome segregation [25], [26], [28], and both proteins have a catalytically active tyrosine residue that are essential for meiotic DSB induction [28]. In contrast, both AtSpo11-3 and AtTopVIB function in somatic endoreduplication [29], [30], [31]. AtSpo11-3 and AtTopVIB interact in the yeast two-hybrid system and may form a putative TopVI complex with RHL1, BIN4, MID or other components [32], [33], [34]. Proteins corresponding to AtSpo11-1, AtSpo11-2, AtSpo11-3 and AtTopVIB also exist in *O. sativa*, which are named OsTop6A1, OsTop6A2, OsTop6A3 and OsTop6B, respectively in *indica* rice [23], and named OsSpo11-1 [35], OsSpo11-2, OsSpo11-3 and OsTopVIB, respectively in *japonica* rice to follow the nomenclature of their homologues in other eukaryotes (in this article, eukaryotic members of the TopVIA/Spo11 family are indicated by terms containing “Spo11” except for the above three members in *indica* rice; whereas “TopVIA” indicates the archaeal member). Yeast two-hybrid assay of proteins from *indica* rice showed that OsTop6B interacts with both OsTop6A2 and OsTop6A3 but not with OsTop6A1 [23]. Overexpression of *OsTOP6A1*, *OsTOP6A3* or *OsTOP6B* in *Arabidopsis* results in reduced sensitivity to abscisic acid and enhanced tolerance to high salinity and dehydration [23], [36], indicating that they might have a function in stress tolerance. Recently, RNAi analysis of *OsSPO11-1* reveals that this gene is required for homologous pairing, recombination, synaptonemal complex installation and crossover formation in meiosis [35].

In this study, we identified two novel Spo11/TopVIA homologues (named OsSpo11-4 and OsSpo11-5, respectively) from the model monocot plant rice (*O. sativa* L. ssp. *japonica*). We observed the interaction of OsSpo11-4 and OsTopVIB by both yeast two-hybrid and pull-down assays. We found that OsSpo11-4, rather than OsSpo11-1 or OsSpo11-5, was able to cleave double-strand DNA (dsDNA) *in vitro*. This is the first *in vitro* enzymatic evidence that a plant Spo11/TopVIA homologue has double-strand DNA cleavage activity. We further analyzed the function of OsSpo11-4 in growth and development using RNAi approach and showed that OsSpo11-4 was essential for meiosis.

## Results

### Identification of *SPO11* and *TOPVIB* homologues in rice

Previous studies have identified three TopVIA/Spo11 homologues in rice, namely OsTop6A1/OsSpo11-1, OsTop6A2/OsSpo11-2, and OsTop6A3/OsSpo11-3 [23], [35]. Here, using amino acid sequences of the yeast Spo11 protein as query to search the rice genome sequence in TIGR (http://rice.plantbiology.msu.edu/) with the TBLASTN program, we identified 3 proteins homologous to Spo11 in *japonica* rice: one is the identified OsSpo11-1 protein (NCBI accession number: GU170363); and the other two are novel proteins which were designated OsSpo11-4 (GU177866) and OsSpo11-5 (GU170364) respectively, as putative members of the Spo11/TopVIA family. The full-length cDNAs of OsSpo11-4 and OsSpo11-5 were isolated by RT-PCR and RACE, using gene-specific primers. The genomic sequences of the 2 novel genes were further examined by BLASTN searches of the TIGR rice genomic database with respective full-length cDNA used as queries. Each of the 2 genes is present in the rice genome as a single-copy gene (*OsSPO11-4*: Os12g0622500 and *OsSPO11-5*: Os11g0545300). Comparison of cDNAs with genomic DNA sequences showed that *OsSPO11-5* consists of 11 introns and 12 exons, with the largest open reading frame (ORF) of 2145 bp encoding 714 putative amino acids, whereas *OsSPO11-4* contains only 1 intron and encodes a predicted protein consisting of 487 amino acids (Figure S1). Further searches of public database using the two genes as queries showed that *OsSPO11-4* and *OsSPO11-5* are also present in the genome of *indica* rice (EAY83148 and EEC68321, respectively) but absent in completely sequenced genomes of other plants that include dicots such as *Arabidopsis* and monocots such as maize, indicating that both genes exist just in rice genome.

**Figure 1:**
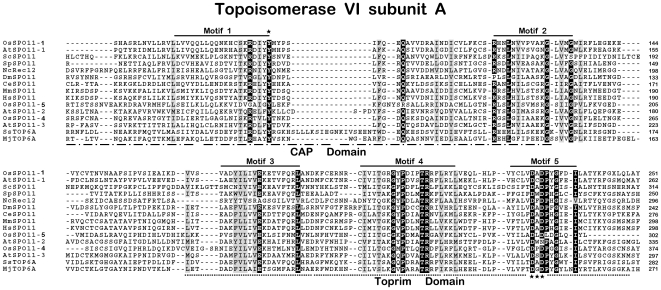
Multiple alignment of amino acid sequences of OsSpo11s and their homologues using ClustalX software (version 1.8). Gaps are shown by dashes. Black boxes indicate conserved residues, and grey boxes indicate similar residues. The respective amino acid position of each sequence is given on the right. The active tyrosine residue and the DXD sequence identified in archaeal TopVIA are marked with asterisks in motif I and motif V, respectively. Sequences used here are OsSpo11-5C (accession No. AY154916), OsSpo11-1 (GU170363) and OsSpo11-4 (GU177866) from *Oryza sativa*; AtSpo11-1 (AJ251989), AtSpo11-2 (AJ251990) and AtSpo11-3 (AL162973) from *Arabidopsis thaliana*; ScSpo11 (P23179) from *Saccharomyces cerevisiae*; SpRec12 (P40384) from *Schizosaccharomyces pombe*; NcSpo11 (CAB88597) from *Neurospora crassa*; DmSpo11 (AAC61735) from *Drosophila melanogaster*; CeSpo11 (CAA92974) from *Caenorhabditis elegans*; MmSpo11 (Q9WTK8) from *Mus musculus*; HsSpo11 (Q9Y5K1) from *Homo sapiens*; SsTopVIA (O05208) from *Sulfolobus shibatae* and MjTopVIA (Q57815) from *Methanobacterium janaschii*.

OsSpo11-4 and OsSpo11-5 shared only 15.9∼24.2% similarity with the three identified Spo11/TopVIA homologues in rice (OsTop6A1/OsSpo11-1, OsTop6A2/OsSpo11-2, and OsTop6A3/OsSpo11-3). Multiple sequence alignment of the 2 novel OsSpo11s with other Spo11/TopVIA sequences revealed that similarity mainly occurred in the 5 conserved motifs initially identified in archaeal TopVIA [4] (Figure 1). For OsSpo11-5, these conserved motifs distributed within its C-terminal part of 302 amino acids (OsSpo11-5C) and its N-terminal extension of 412 amino acids (OsSop11-5N) was functionally unknown. The proposed active site tyrosine in motif I, which is responsible for the formation of double-strand breaks in yeast and invariant in all known members of the Spo11/TopVIA family, such as Tyr^103^ in MjTopVIA, and Tyr^135^ in ScSpo11 [3], [4], [11], was conserved in OsSpo11-1 and the 2 novel OsSpo11s (Tyr^92^ in OsSpo11-1, Tyr^213^ in OsSpo11-4, and Tyr^152^ in OsSpo11-5C). Furthermore, all identified Spo11/TopVIA members contain 3 invariant acidic amino acids in motifs III and V [3]; and in this conserved acidic group, two Asp residues constitute a DXD sequence in motif V, which is proposed to coordinate divalent metals, especially Mg^2+^ ion [1], [3]. In yeast, a mutation in this acidic group impairs or abolishes the ability of Spo11 to generate DSBs *in vivo* [3]. The 3 conserved acidic amino acids were also conserved in the 3 OsSpo11s (Glu^178^, Asp^229^ and Asp^231^ of OsSpo11-1, Glu^301^, Asp^352^ and Asp^354^ of OsSpo11-4, and Glu^200^ in motif III, Asp^292^ and Asp^294^ in motif V of OsSpo11-5C) (Figure 1). These results suggest that like OsSpo11-1, OsSpo11-4 and OsSpo11-5 are also homologues of Spo11/TopVIA.

**Figure 2:**
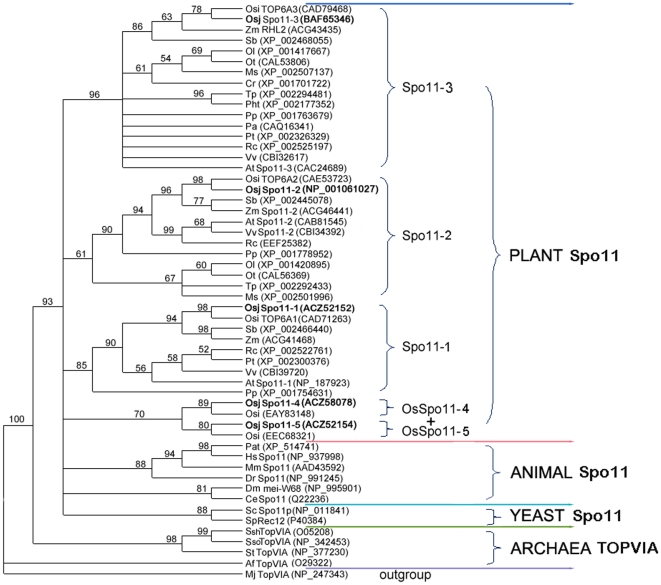
Unrooted Maximum Likelihood tree of Spo11/TopVIA homologues. Sequences were obtained from the ncbi database (http://www.ncbi.nlm.nih.gov/) using the BLASTP or TBLASTN with OsSpo11-1∼5 used as quiries, and amino acids representing CAP and Toprim domain were aligned by Clustal X (version 1.8). A tree with 54 unrepeated sequences, based on a preliminary exhaustive analysis, the unambiguously aligned positions were used for tree calculation by Phyml and a JJT model of amino acid substitution. The robustness of each branch was evaluated by non-parametric bootstrap analysis for 100 replicates using Phyml software. Only bootstrap values higher than 50% are shown. MjTopVIA was selected as outgroup. Os: *Oryza sativa*; Zm: *Zea mays*; Sb: *Sorghum bicolor*; Ol: *Ostreococcus lucimarinus*; Ot: *Ostreococcus tauri*; Ms: *Micromonas*; Cr: *Chlamydomonas reinhardtii*; Tp: *thalassiosira pseudonana*; Pht: *Phaeodactylum tricornutum*; Pp; *Physcomitrella patens*; Pa: *Pyrococcus abyssi*; Pt: *Populus trichocarpa*; Rc: *Ricinus communis*; Vv: *Vitis vinifera*; At: *Arabidopsis thaliana*; Pat: *Pan troglodytes*; Hs: *Homo sapiens*; Mm: *Mus musculus*; Dr: *Danio rerio*; Dm: *Drosophila melanogaster*; Ce: *Caenorhabditis elegans*; Sc: *Saccharomyces cerevisiae*; Sp: *Schizosaccharomyces pombe*; Ssh: *Sulfolobus shibatae*; Sso: Sulfolobus solfataricus; St: *Sulfolobus tokodaii*; Af: *Archaeoglobus fulgidus*; Mj: *Methanobacterium janaschii*.

In order to analyze the evolutional relationship of Spo11/TopVIA homologues, members of this family were identified from plants, animals, fungi and representatives of archaea using Blast searches against public database. Phylogenetic analyses showed that plant Spo11 homologues fell into 4 distinct groups, which are respectively represented by Spo11-1, Spo11-2 and Spo11-3 in *Arabidopsis* and rice, and OsSpo11-5&OsSpo11-4 (Figure 2). The five OsSpo11 proteins are more closely homologous to their corresponding Spo11/TopVIA members from other organisms than to each other except for OsSpo11-5 with OsSpo11-4. Moreover, it has been known that Spo11-1 and Spo11-2 are involved in meiosis, Spo11-3 in endoreduplication, while functions of OsSpo11-5 and OsSpo11-4 are unclear previously. Therefore, the five *SPO11* genes in rice might not arise through recent duplication events, but represent different ancient paralogues.

The identified *OsTOP6B* of *indica* rice also exist in *japonica* rice (AY371050), which is named *OsTOPVIB* in our study. OsTopVIB contained the 4 motifs (B1 to B4) conserved in TopVIB proteins from other organisms [23] (Figure S2). In archaeal TopVIB, Asn in motif B1 (Asn^42^ in SsTopVIB) is identified as Mg^2+^ binding residue and Asp and Gly residues in motif B2 are involved in nucleotide contacts (Asp^76^ and Gly^80^ in SshTopVIB). These residues are part of the GHKL domains involved in ATP binding and the nucleotide-binding pocket [8]. Our analysis showed that these residues were conserved in the rice TopVIB protein (Asn^93^, Asp^187^ and Gly^191^ in OsTopVIB, respectively). The middle region of OsTopVIB matched with the helix-two turns-helix domain characterized by 9- to 12-amino acid insertion peptides, which functions as a linker to position the N- and C-terminal domains in archaea [8]. A postulated motif B4 was identified in OsTopVIB; this motif B4 in archaeal TopVIB is related to the ATPase domain of GyrB, which is referred to as a transducer domain and has been proposed to mediate intersubunit communication by structurally transforming signals from the ATP binding site of the GHKL domain to the DNA binding and cleavage domains of the holoenzyme [2], [8].

### OsSpo11-4 interacts with OsTopVIB

**Figure 3:**
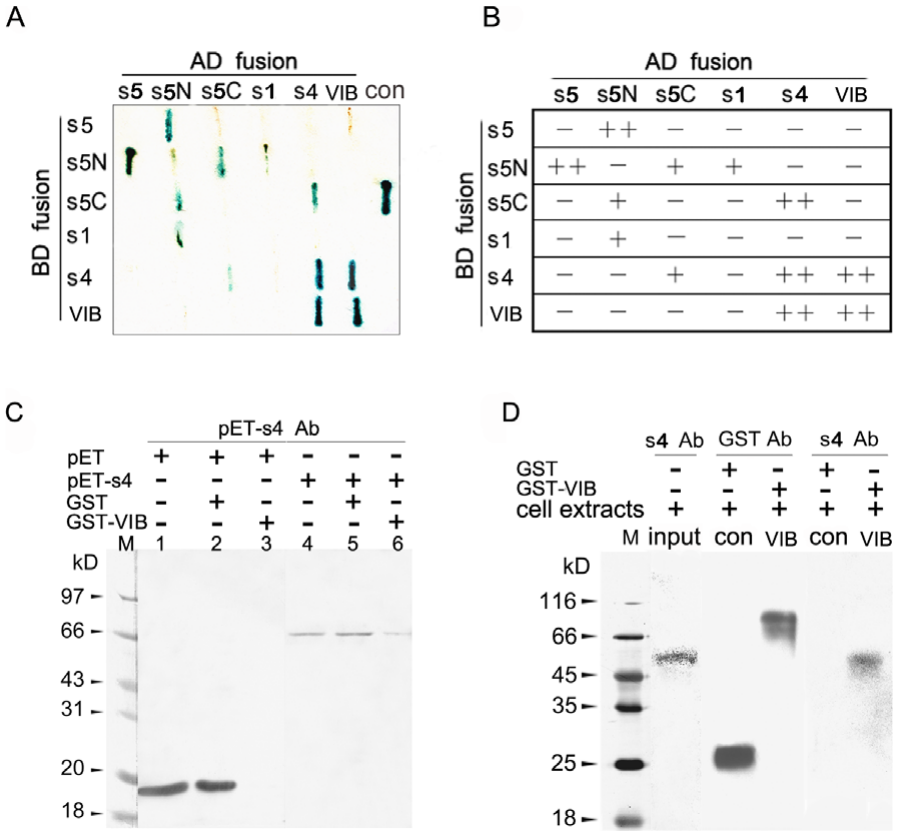
Interactions of OsSpo11 and OsTopVIB proteins. **A** and **B**, Yeast two-hybrid assay. Different dual-combinations of prey (AD-fusion) and bait (BD-fusion) constructs were cotransformed into yeast strain AH109. **A**, X-gal staining results and a comparison with the positive control (con) supplied by the Yeast Two Hybrid Kit. These colonies were first screened by growth on QDO (SD/-Ade/-His/-Leu/-Trp) medium lacking adenine, histidine, leucine and tryptophan, and then analyzed by X-gal staining for 7 h. **B**, Interaction evaluated according to X-gal staining results: strong (++), weak (+) or no (−) interaction. s5, OsSpo11-5; s5N, N-extension of 412 amino acids of OsSpo11-5; s5C, C-terminal 302AAs TopVIA region of OsSpo11-5; s1, OsSpo11-1; s4, OsSpo11-4; VIB, OsTopVIB. **C** and **D**, Pull-down assay. **C**, Purified pET-OsSpo11-4 or pET tag (from pET32a vector) expressed in *E. coli* incubated with GST-OsTopVIB-bound resin. The pulldowns were examined by western blot analysis with an antibody against pET-Spo11-4. Lane 1, purified pET tag alone. Lane 2, precleared pET tag using GST-binding resin. Lane 3, resin-bound GST-OsTopVIB incubated with precleared pET tag. Lane 4, purified pET-OsSpo11-4 fusion protein alone. Lane 5, precleared pET-OsSpo11-4 fusion protein using GST-binding resin. Lane 6, resin-bound GST-OsTopVIB incubated with precleared pET-OsSpo11-4 fusion protein. **D**, Proteins extracted from rice flowers at the male meiosis stage incubated with resin-bound GST (con) or GST-OsTopVIB (VIB). The pulldowns were subjected to SDS-PAGE and then Western blot analysis with an antibody against GST (GST Ab) or OsSpo11-4 (S4 Ab).

Considering that archaeal TopVI functions as an A_2_B_2_ heterotetramer and rice genome encodes Spo11/TopVIA and TopVIB homologs, we first examined interactions among OsSpo11s and OsTopVIB using yeast two-hybrid assay. The results demonstrated that both OsSpo11-4 and OsTopVIB can strongly self-interact, which suggests that each may form a homodimer. OsSpo11-1 had no detectable self-interaction. OsSpo11-5 had undetectable self-interaction, but the N-terminal extension sequence (OsSpo11-5N) of OsSpo11-5 can strongly interact with the full-length protein and relatively weakly with the C-terminal part (OsSpo11-5C) of the protein. In addition, relatively weak interactions were detected in OsSpo11-5N/OsSpo11-1 and OsSpo11-5C/OsSpo11-4 (Figure 3A and B). Importantly, this analysis revealed that OsTopVIB interacts with OsSpo11-4 strongly but not with OsSpo11-5 and OsSpo11-1.

We further validated the interaction between OsSpo11-4 and OsTopVIB by GST pull-down assay. The purified pET-OsSpo11-4 (purified under native conditions) and pET tag (from pET-32a vector, control) were precleared with GST preabsorbed in GST affinity resin, then yeast expressed, GST affinity resin-binding GST-OsTopVIB fusion protein was incubated with the precleared pET-OsSpo11-4 or pET tag. Western blot analysis revealed that pET-OsSpo11-4 (about 72 kD) could interact with GST-OsTopVIB (Figure 3C).

We also examined the interaction of OsSpo11-4 and OsTopVIB using purified OsTopVIB proteins and cell protein extracts from rice flowers (Figure 3D). The OsTopVIB-GST fusion or GST (control) was incubated with protein extracts from flowers at the meiosis stage. After the resulting resin was washed to remove unspecified proteins, specifically bound proteins were analyzed by SDS-PAGE and western blotting. An antibody against GST detected the GST band (about 26 kD) in GST pulldowns and detected the GST-OsTopVIB band (about 103 kD) in GST-OsTopVIB pulldowns, which indicates that GST and GST-OsTopVIB, respectively, were bound to the GST affinity resin. The antibody against OsSpo11-4 detected the OsSpo11-4 band (55 kDa) in the GST-OsTopVIB pulldowns but not in the GST control (Figure 3D). These results indicated possible interaction of OsTopVIB and OsSpo11-4 *in vivo*. In short, the yeast two-hybrid and pull-down results suggest that OsSpo11-4 and OsTopVIB may interact to form a functional complex *in vivo*.

### OsSpo11-4 is able to catalyze double-strand DNA cleavage *in vitro*

**Figure 4:**
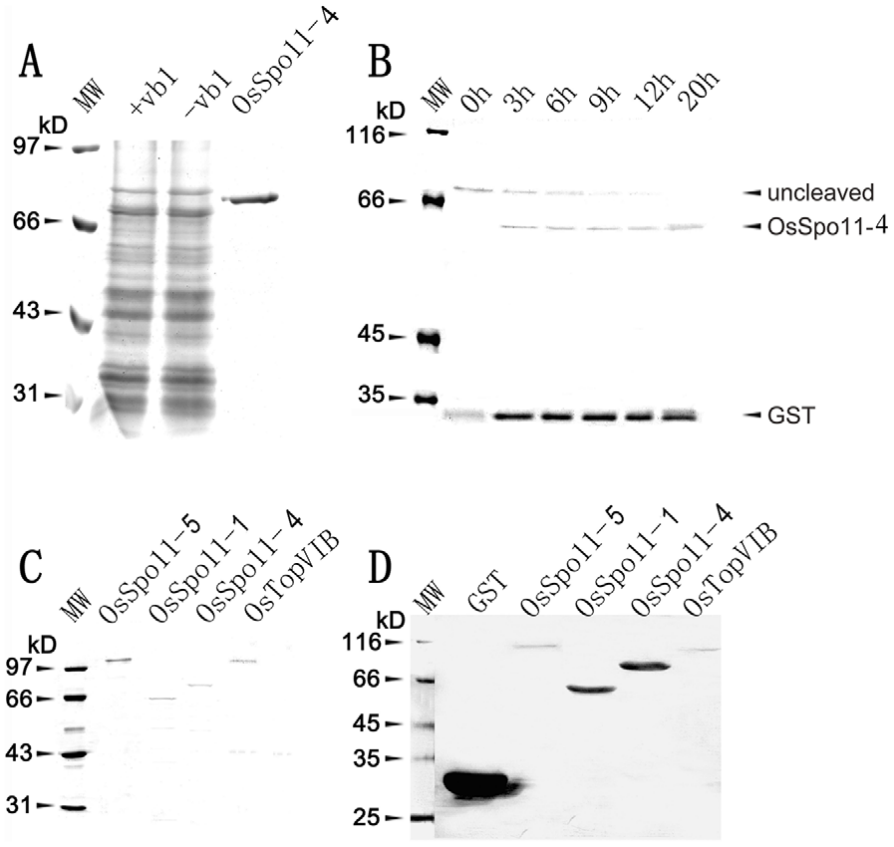
Expression and purification of OsSpo11 and OsTopVIB proteins. Proteins were separated by 10% SDS-PAGE and stained with Coomassie Brilliant Blue (A∼C) or hybridized with an antibody against GST (D). **A**, Expression of the 4 proteins each in *S. prombe* cells (OsSpo11-4 showed here as an example). Cells harboring recombinant plasmids were treated with (+vb1) or without (−vb1) vitamin B1. GST-OsSpo11-4 fusion protein from −vb1 was purified with Glutathione Sepharose 4B resin. **B**, Removal of GST tag from purified GST-protein fusion by thrombin digestion (OsSpo11-4 shown as an example). The reaction mixture was incubated for different times shown above the image and subjected to SDS-PAGE separation. **C**, Purified GST-tagged OsSpo11s and OsTopVIB. **D**, Western blot analysis of purified GST-tagged proteins in C with a monoclonal antibody against GST. MW, molecular weight standards.

**Figure 5:**
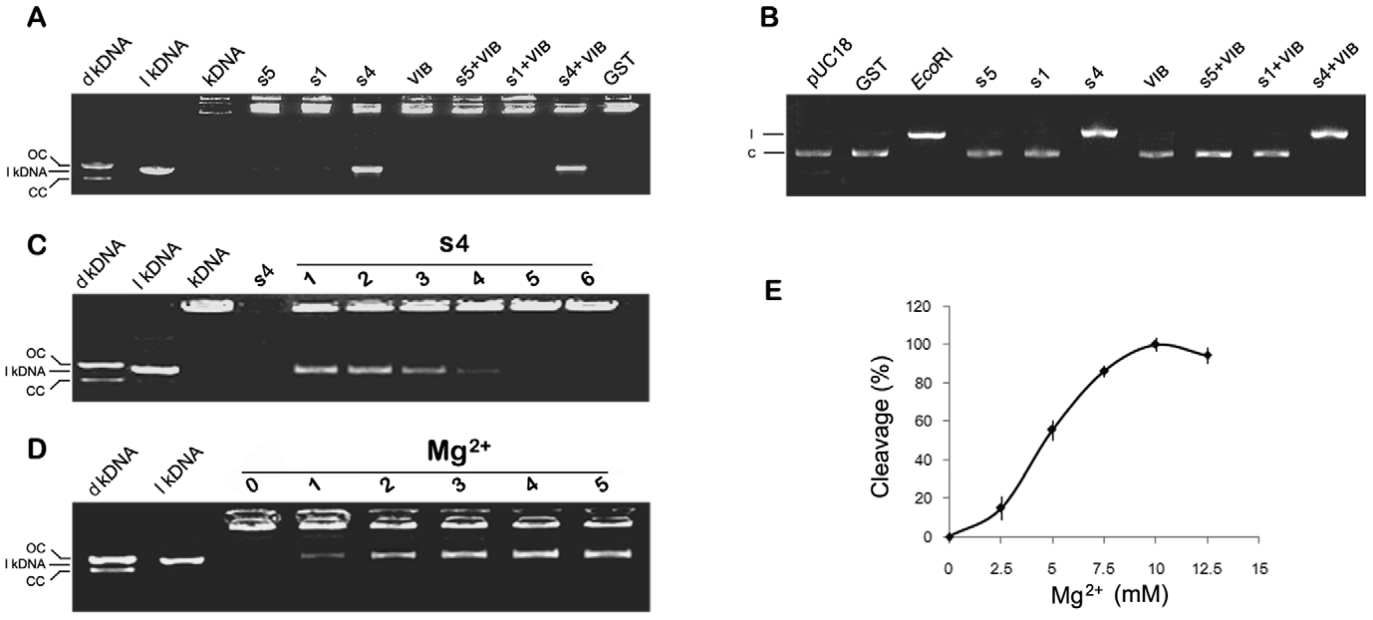
Double-strand DNA cleavage catalyzed by purified OsSpo11 and OsTopVIB proteins. Each purified OsSpo11 and OsTopVIB protein or a combination shown above the image was added into a reaction mixture containing substrate, and purified GST was used as a control. After reaction, the mixture was subjected to agarose separation (for details, see “Materials and methods”). dkDNA, decatenated kDNA Marker; lkDNA, linear kDNA marker; kDNA, kinetoplast DNA; s5, OsSpo11-5; s1, OsSpo11-1; s4, OsSpo11-4; VIB, TopVIB; GST, the GST protein control. **A**, kDNA as a substrate. dkDNA shows relative positions of open circular nicked DNA—OC, and relaxed, closed circular monomers—CC; dkDNA and lkDNA markers were provided by the Topoisomerase Assay Kit, kDNA was used as a catenated DNA reference after incubation in a reaction mixture without protein. **B**, pUC18 plasmid as a substrate. pUC18 refers to a reaction containing buffer and pUC18 plasmids only (c, circular pUC18 marker). *Eco*RI refers to pUC18 plasmids digested by *Eco*RI, which cuts pUC18 only once (l, linear pUC18 marker). **C**, DNA cleavage reaction rate is proportioned to the concentration of OsSpo11-4. s4 refers to only reaction buffer and OsSpo11-4 were added, while 1∼6 refer to 0.5 µM, 0.4 µM, 0.3 µM, 0.2 µM, 0.1 µM and 0 µM OsSpo11-4 were added to the standard reaction, respectively. Cleavage activity was determined using kDNA decatenation assays. **D**, the effect of Mg^2+^ on OsSpo11-4 activity. 0∼5 refer to 0 mM, 2.5 mM, 5 mM, 7.5 mM, 10 mM and 12.5 mM Mg^2^ were added to the standard reaction (kDNA decatenation), respectively. **E**, reaction rate quantification of 0∼5 in panel D. Reaction rate was determined as a percentage of linear kDNA generated compared to the total kDNA added.

To address the enzymatic properties of OsSpo11s and OsTopVIB, we used a eukaryotic yeast expression system for generation of soluble proteins that are often critical for the structure and activity of eukaryotic proteins. We purified OsSpo11-1, OsSpo11-4, OsSpo11-5 and OsTopVIB by GST affinity chromatography (Figure 4A, 4C and 4D). These native proteins were further collected by removing the GST tag (Figure 4B). The purified native OsSpo11-1, OsSpo11-4, OsSpo11-5 and OsTopVIB proteins were incubated with kDNA to assay the activity of decatenation, which is specifically catalyzed by type II DNA topoisomerase. kDNA is a catenated DNA extracted from the kinetoplast of insect trypanosome *Crithidia fasciculate*, which is composed of the aggregation of interlocked DNA circles with high molecular size. These high-molecular-size networks cannot migrate from the loading pore. When the networks were cleaved, minicircular DNAs (cleaved and resealed) or linear DNAs (lkDNA, unresealed) were released and quickly moved into the gel. As shown in Figure 5A, the decatenated kDNA marker (dkDNA, prepared by decatenation reaction catalyzed by topoisomerase II) showed relative positions of open circular nicked DNA (OC) and closed circular monomers (CC). Among the examined proteins, only OsSpo11-4 could catalyze the catenated kDNAs into free linear forms completely, which have a molecular size equal to that of the lkDNA marker. Moreover, the decatenation reaction rate was proportioned to the concentration of OsSpo11-4 (Figure 5C), these results suggest that OsSpo11-4 can specifically cleave kDNAs to produce double-strand breaks. OsSpo11-4 had the enzymatic activity alone and appeared to be independent of OsTopVIB. This finding is inconsistent with archaeal TopVI, in which catalytic subunit TopVIA performs decatenation of tangled DNA in the presence of the B subunit [1], [2]. Furthermore, when kDNA was decatenated by archaeal (*S. shibatae*) TopVI, open circular and relaxed, covalently closed circular DNA rings were produced, which indicates that archaeal TopVI can re-ligate the broken dsDNA ends to covalently closed DNA rings [1], [2], whereas OsSpo11-4 appeared not to have this activity.

We also examined the cleavage activity of these proteins using the pUC18 plasmid as reaction substrate. Consistent with the above data, only OsSpo11-4 had the activity to cleave pUC18 plasmids to linear DNAs, and other proteins had no detectable activity (Figure 5B). Together, these results clearly indicate that OsSpo11-4 itself can cleave dsDNA, and its enzymatic activity appears independent of OsTopVIB.

Although the DNA cleavage activity of OsSpo11-4 was independent of OsTopVIB, it was strictly Mg^2+^-dependent. OsSpo11-4 created DSBs on kDNA substrates only in the presence of Mg^2+^, which leads to accumulation of linearized kDNA (Figure 5D). The reaction rate enhanced when the concentration of the Mg^2+^ increased up to 10 mM; however, concentrations of Mg^2+^ higher than 10 mM did not further increase the amount of kDNA, on the contrary, the cleavage activity of OsSpo11-4 declined when exposed to higher levels of Mg^2+^(Figure 5E).

### *OsSPO11-4* is expressed preferentially in flowers

**Figure 6:**
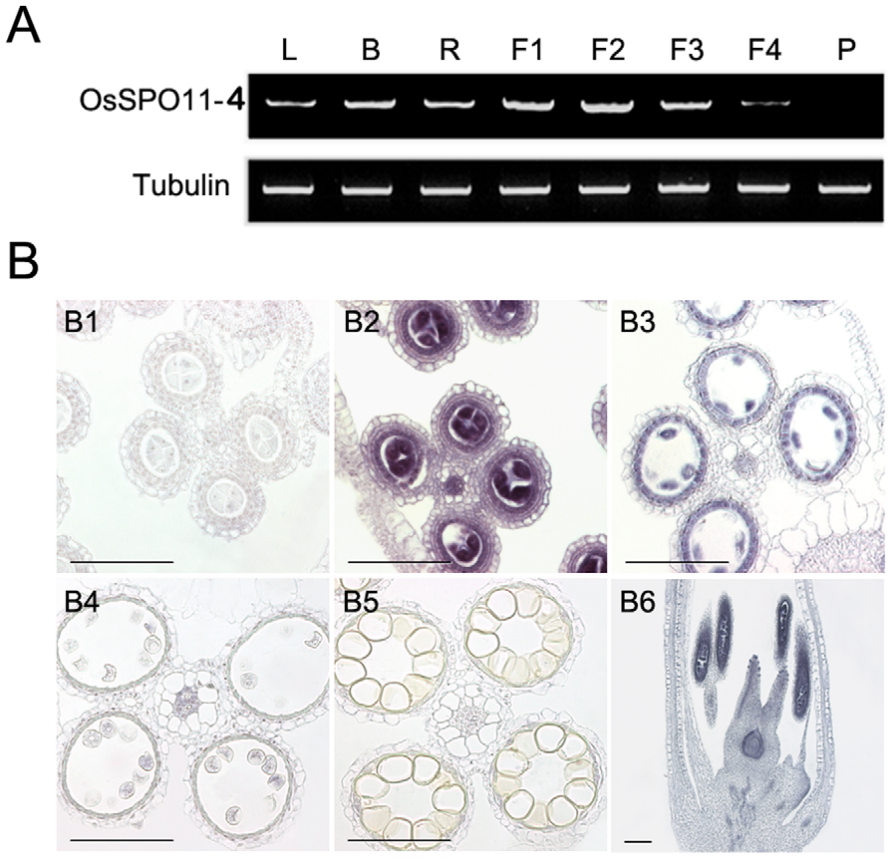
Accumulation patterns of OsSpo11-4 mRNA in different organs. **A**, Semi-quantitative RT-PCR analysis of OsSpo11-4 mRNA in vegetative tissues and flowers at different stages. L, leaves from two-week-old seedlings; B, young buds; R, young roots; F1∼F4, flowers at stamen and carpel primordial formation stage (F1), pollen mother cell formation stage (F2), male meiosis stage (F3) and uninucleate microspore stage (F4); P, pollen grains. TubA mRNA was amplified as an internal standard. **B**, *in situ* analysis of OsSpo11-4 mRNA with DIG-labeled antisense (B2∼B6) or sense probes (B1) in developing flowers. Hybridization was carried out on transverse sections of wild-type flowers at the pollen mother cell stage (B1 and B2), meiosis stage (B3), uninucleate microspore stage (B4) and prepollinated flower stage (B5), or on vertical cuts of wild-type flowers at the meiosis stage (B6). Scale bar = 50 µm in B1∼B5, and 200 µm in B6.

Semi-quantitative RT-PCR was used to evaluate mRNA levels of *OsSPO11-4* in different rice tissues, including 2-week-old leaves, young roots, young shoots and flowers at different meiotic stages. *OsSPO11-4* was expressed at the highest level in flowers, in which pollen mother cells were at the meiotic phase, and at relatively lower levels in roots, buds and leaves, with no detectable expression in mature pollen grains (Figure 6A). The levels of OsSPO11-4 mRNA appeared to be developmentally dependent in flowers, showing the highest levels at the pre-meiotic and meiotic stages, and the gradually decreased levels with advancing development (Figure 6A).

We further used RNA *in situ* hybridization to examine the temporal and spatial expression patterns of *OsSPO11-4* in flowers using DIG-labeled antisense and sense (control) probes. In flowers, the OsSpo11-4 antisense probe detected strong signals in pre-meiotic and meiotic pollen mother cells, tapetal cells and meiotic ovaries. No signals were detected in spores at the uninucleate microspore and subsequent stages or in other flower organs such as lemma and palea. Sense probes did not detect signals (Figure 6B). The expression pattern suggests that OsSpo11-4 might function in pre-meiotic and meiotic pollen mother cells.

### OsSpo11-4 is required for efficient meiosis

**Figure 7:**
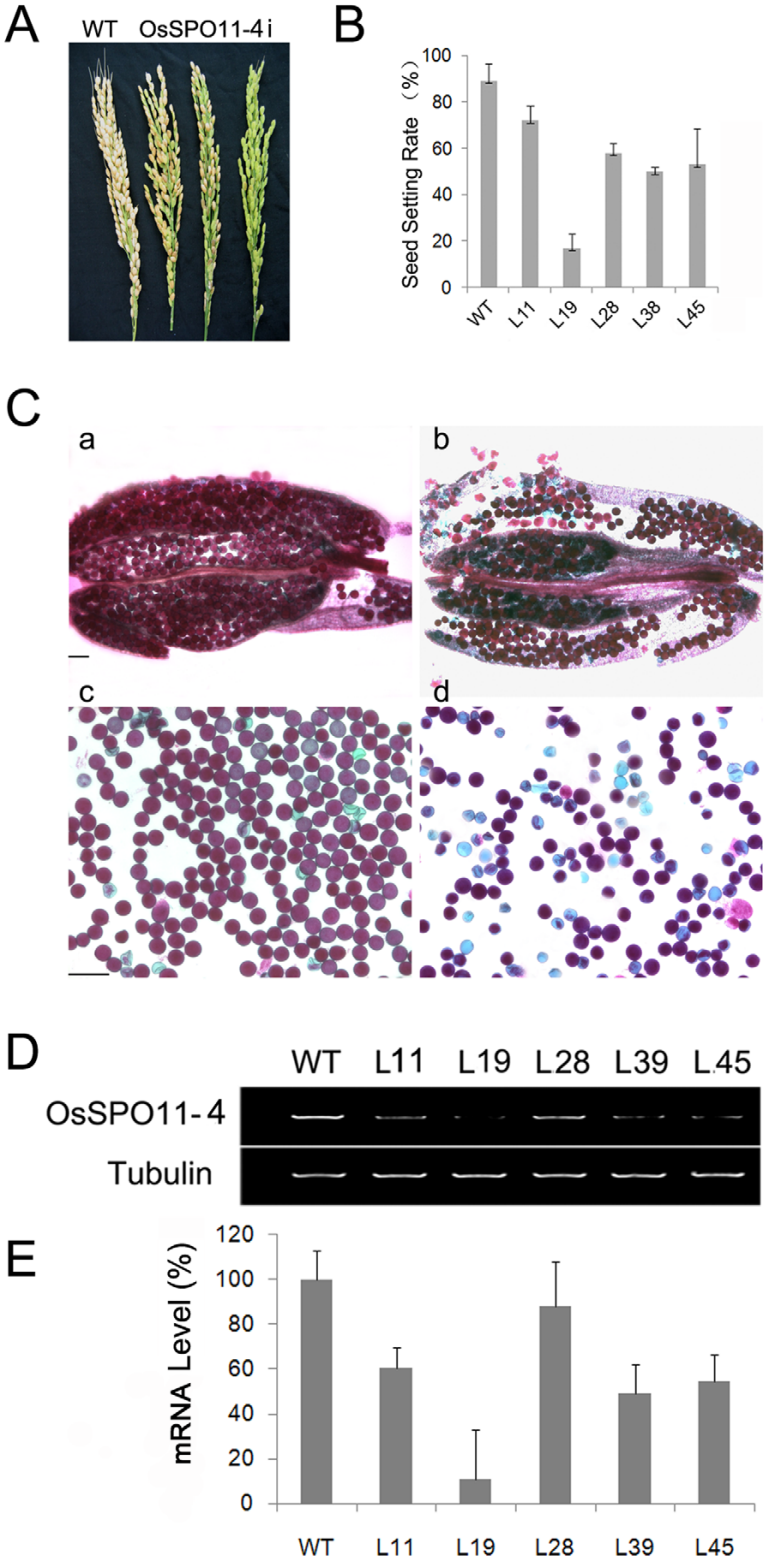
Seed setting (A, B), pollen viability (C) and endogenous OsSpo11-4 expression (D) were decreased in OsSpo11-4 RNAi lines. **A**, Panicle morphology of wild-type (left) and OsSpo11-4 RNAi T0 lines with different seed setting (right 3). **B**, Seed setting of wild-type (WT) and 6 RNAi T1 generation lines (L11, L19, L8, L38, L39 and L45). **C**, Alexander staining of anthers (a and b) and mature pollen grains (c and d) from wild-type (a and c) and RNAi (b and d) plants showing reduced pollen viability in RNAi lines. Scale bar = 100 µm in (a) and (b), and 50 µm in (c) and (d). **D**, Semi-quantitative RT-PCR analysis of endogenous OsSpo11-4 mRNA levels in wild-type and RNAi lines. TubA mRNA was amplified as an internal control. All PCR products were separated on 1% agarose gel. **E**, Quantitative real-time RT-PCR analysis for expression of OsSpo11-4 in wild type and RNAi lines. The expression levels of SPO11-4 in different RNAi lines were firstly normalized by computing to the internal standard gene, *tubA*, and then compared to the wild type by the ΔΔC_T_ method.

To address the *in vivo* function of OsSpo11-4, we obtained RNAi lines of *OsSpo11-4* using gene-specific cDNA fragments. The presence of the transgene in hygromycin-resistant rice planets was examined by PCR with primer pairs localized to the spacer and inserted cDNA sequences on the OsSPO11i vector. The PCR-positive transgenic lines showed decreased seed setting rate to different degrees as compared with wild-type plants (Figure 7A). Furthermore, 6 lines representing different sterile phenotypes (L11, L19, L28, L39, L39, L45), identified to have a single-copy insertion of the transgene by Southern blot hybridization, were used to generate T1 plants. T1 generations showed stable and heritable sterile phenotypes (17% seed setting rate in one line, ranging from 50% to 58% for 4 lines and 72% in 1 line, with 91% in the wild type) (Figure 7B). The endogenous transcripts of *OsSPO11-4* were considerably downregulated in OsSpo11-4i lines as compared with the wild-type control (Figure 7D). To evaluate whether the sterile phenotype involved pollen abortion, we examined the viability of pollen grains from OsSpo11-4i lines at maturity using Alexander staining, whereby viable pollen grains are stained red and non-viable ones stained green [37] (Figure 7C). Most of the wild-type pollen grains (96%, n = 1525) were viable, whereas a high proportion of pollen grains from OsSpo11-4i lines were non-viable (31∼76% variable in different lines), which suggests that pollen abortion in OsSpo11-4i lines related to the sterile phenotype.

**Figure 8:**
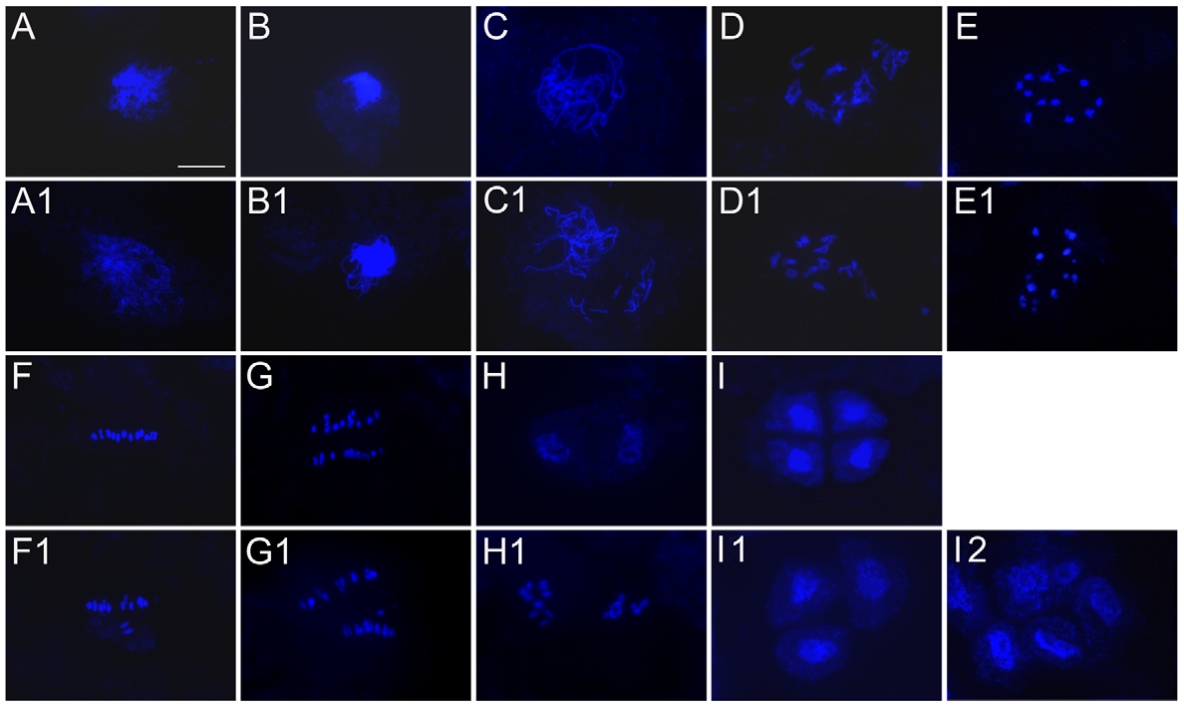
Male meiosis in wild-type and OsSpo11-4i plants. Male nuclear spreads were prepared from wild type (A∼I) and OsSpo11-4i (A1∼I2) plants and stained with DAPI. **A** and **A1**, leptotene; **B** and **B1**, early zygotene; **C** and **C1**, pachytene; **D** and **D1**, diplotene; **E** and **E1**, diakinesis; **F** and **F1**, metaphase I; **G** and **G1**, anaphase I; **H** and **H1**, telophase I; **I**, **I1** and **I2**, tetrad stage (**I**: normal tetrad, **I1**: triad, **I2**: polyad). Scale bar = 10 µm in A for A∼I and A1∼J1.

To determine whether the sterile pollen grains resulted from meiotic defects in RNAi plants, we examined the meiotic chromosome behavior using DAPI-stained chromosome spreads of male meiocytes from the RNAi line L19, along with the wild-type control (Figure 8). In wild-type plants (Figure 8A-I), chromosomes in leptotene cells appear as thin threads. Homologous chromosomes begin to associate side by side at zygotene and fully synapse and condense into thick threads at pachytene. Synapsed homologous chromosomes begin to separate at diplotene, and 12 bivalents become highly condensed at diplotene and diakinesis. Thereafter, 12 highly condensed bivalents align on equator plates at metaphase I and are subject to reductional division at anaphase I. The segregated univalents in each pole were partially decondensed at telophase I, finally generating dyads. During meiosis II, the 2 daughter cells divide simultaneously with parallel orientations of spindles, finally separating to generate 4 haploid tetrads.

Male meiocytes from OsSpo11-4i lines did not display obvious aberrance from premeiotic interphase to middle zygotene (Figure 8A1 and 8B1) as compared with wild-type male meiocytes (Figure 8A and 8B). The detectable defects appeared in pachytene male meiocytes; 20.4% of the examined male meiocytes at this phase (n = 298) had chromosome segments (Figure 8C1). For male meiosis entering into late diplotene and diakinesis, 12 bivalents appeared in wild-type male meiocytes (Figure 8D and 8E); however, 40.7% (n = 452) of OsSpo11-4i male meiocytes had more than 12 distinguishable chromosomes (Figure 8D1 and 8E1), which indicates the presence of univalents or chromosome fragments. At metaphase I, these aberrant chromosomes dispersed throughout the nucleus rather than aligning on the metaphase plate (Figure 8F1); lagging or/and unequally segregated chromosomes were observed at anaphase I (Figure 8G1). A mixture of normal and aberrant dyads existed at telophase I (Figure 8H1). These defects led to aberrant chromosome behavior and unequal separation of chromosomes at meiosis II, generating triads and polyads with variable chromosome contents (Figure 8I1 and 8I2).

## Discussion

The archaeal TopVI, a heterotetramer composed of two A subunits (TopVIA) and two B subunits (TopVIB), has the ability to pass DNA double strands through each other. When the enzymatic reaction occurs, TopVI cleaves one DNA duplex to open a DNA gate and captures another DNA duplex to pass though the gate, then rejoins the two ends of the cleaved DNA duplex to close the gate; this topoisomerase activity enables TopVI to solve topological problems of DNA during replication, transcription, recombination and chromosome segregation [38]. Yeast and currently sequenced animal genomes contain one homologue of TopVIA, namely Spo11, and no homologue of TopVIB [27]. The Spo11 protein maintains the activity of creating double-strand breaks but does not rejoin the DNA breaks after cleavage [39]; Spo11 plays a conserved role in initiation of homologous recombination during meiosis, which requires its cleavage activity [4], [9].

Different from the yeast and animal genomes, the completely sequenced genomes of higher plants have at least three Spo11/TopVIA homologues and one TopVIB homologue [27]. Previous studies have revealed three Spo11/TopVIA homologues in *Arabidopsis* [21], [22] and their corresponding proteins in rice [23]. In this study, we identified two novel Spo11/TopVIA homologues (OsSpo11-4 and OsSpo11-5), which exist only in the rice genome. Therefore there are at least five Spo11/TopVIA homologues in rice. These plant Spo11/TopVIA homologues share low similarity with each other (20∼30%), suggesting they might be different in functions. Genetic and functional analysis of the three Spo11/TopVIA homologues in *Arabidopsis* have demonstrated that AtSpo11-1 and AtSpo11-2 have a function similar to the yeast Spo11 protein [25], [26], [28] whereas AtSpo11-3 may interact with AtTopVIB to form a putative TopVI complex and functions in somatic endoreduplication [29], [30], [31]. The recent study in rice shows that OsSpo11-1 is necessary for meiotic pairing and crossover formation, indicating that Spo11-1 plays a conserved role in *Arabidopsis* and rice [35]. The function of Spo11-2 and Spo11-3 might also be highly conserved among plants; however, since OsSpo11-4 and OsSpo11-5 are present just in rice, they may have roles distinct from Spo11-1, Spo11-2 and Spo11-3.

Our enzyme activity analysis showed that OsSpo11-4 itself can catalyze DNA cleavage *in vitro* without the help of OsTopVIB and OsSpo11-4 had no detectable activity in resealing the broken ends *in vitro*, with or without OsTopVIB. This *in vitro* enzymatic activity of OsSpo11-4 is distinct from that of archaeal TopVIA, which cleaves DNA only in the presence of TopVIB and reseals the broken DNA ends after cleavage [1]; but it is similar to that of the Spo11 protein from yeast and animals, which produce double-strand breaks in the absence of TopVIB and does not rejoin DNA breaks [39]. It is interesting that although OsSpo11-4 is more analogous to Spo11 than to TopVIA in terms of *in vitro* enzymatic features, it interacts with OsTopVIB, which is similar to TopVIA but different from Spo11. Our yeast two-hybrid assay revealed that both OsSpo11-4 and OsTopVIB can self-interact, and OsTopVIB interacts only with OsSpo11-4 among the 3 examined Spo11 proteins (the other two are OsSpo11-1 and OsSpo11-5). Further pull-down assay confirmed the interaction between OsTopVIB and OsSpo11-4. These results indicate that as a TopVIA homologue, OsSpo11-4 might combine with OsTopVIB to form a TopVI heterotetramer similar to that of archaea.

One question raised is whether the function of OsSpo11-4 is more similar to that of yeast and animal Spo11 or to that of archaeal TopVIA. It is possible that the enzymatic activity of OsSpo11-4 *in vivo* is the same as that *in vitro* and OsSpo11-4 plays a role analogous to Spo11, which is creating double-strand breaks to initiate meiotic recombination; as an OsSpo11-4 interactive protein, OsTopVIB might be unnecessary for DNA cleavage but function as an accessory factor similar to Mei4, Ski8/Rec103, Xrs2, Rec102, Rec104, Rec114, Mer2/Rec107, Mre11 and Rad50 in budding yeast, which are essential for other processes during meiotic recombination initiation [40], [41], [42], [43], [44]. Similar to this hypothesis, the *Arabidopsis* Spo11-2 protein, which can interact with AtTopVIB in yeast two-hybrid assay, is required for meiotic recombination [22], [26]. Alternatively, OsSpo11-4 might function as the A subunit of a putative topoisomerase similar to archaeal TopVI, but the full enzymatic activity *in vivo* requires the help of other proteins besides OsTopVIB. Similar to the second hypothesis, in *Arabidopsis*, RHL1, BIN4 and MID have been identified as components of the TopVI complex constituted by AtSpo11-3 and AtTopVIB on the basis of their interaction with AtSpo11-3 [32], [33], [34].

Our cytological analysis demonstrated that downregulated OsSpo11-4 mediated by RNAi led to aberrant meiosis in rice and a proportion of the RNAi meiocytes showed chromosome fragmentation from the pachytene stage. During meiotic recombination, double-strand breaks are generated by Spo11 and then processed and repaired by Rad51, Dmc1 and other accessory proteins [45], [46], thus chromosome fragmentation may result from failure to repair DSBs rather than from failure to generate DSBs. Chromosome fragmentation has been observed in mutants defective in genes required for DSB repair, for example, *RAD51C* in *Arabidopsis* [47]. Therefore, meiotic chromosome fragmentation caused by knockdown of OsSpo11-4 indicates that OsSpo11-4 is possibly involved in processing or repairing DSBs rather than DSB formation during meiotic recombination, which indirectly supports the second hypothesis. Considering that OsSpo11-4 can catalyze DNA cleavage and interact with OsTopVIB, we propose that OsSpo11-4 and OsTopVIB might form a topoisomerase complex similar to archaeal TopVI and play a role in decatenation or/and solving DNA topological problems arising during meiotic DSB processing or repair. Further analyses of *osspo11-1 osspo11-4* double mutants and identification of components of the putative TopVI complex (consisting OsSpo11-4 and OsTopVIB) will be important for understanding the *in vivo* biochemical and biological functions of the OsSpo11-4 protein. It remains an open question why the two additional Spo11/TopVIA homologues, OsSpo11-4 and OsSpo11-5, are required for rice.

## Materials and Methods

### Plant materials

Seedlings of rice Zhonghua 10 (*O. sativa* L. ssp. *japonica*) were planted as described previously [48]. Flowers at different developmental stages were collected. Young leaves were harvested from 3-week-old plants. Young roots and buds were taken from seedlings germinated on sterile-water-soaked papers.

### Extraction of nucleic acids

Total RNA isolated with use of a Trizol Kit (Invitrogen) was treated with RNase-free DNase I (Takara) to remove any genomic DNA contamination. Genomic DNA was extracted from young leaves by the CTAB method [49].

### cDNA isolation

The full-length cDNAs of OsSpo11-4 and OsSpo11-5 were obtained by RT-PCR and rapid amplification of 5′ and 3′ cDNA ends (RACE) with gene-specific primers as described [50]. The OsSpo11-4 specific primers for 3′RACE were WP69F1 (5′-GGG CTGGATTGAACATCTCG-3′) and WP69F2 (5′-AGCGATCAATGACATCTGCG-3′); and for 5′RACE were WP72R1 (5′-GGAAATCCCTTGTTTGGA-3′) and WP72R2 (5′-CCTGTGTGGTATCTGCACTCC-3′). RT-PCR was used to confirm OsSpo11-4 cDNA by use of the primer pair WP79F (5′-AATTTCTTGCCTCTCGCTCAG-3′) and WP79R (5′-CAGACAGAAAGGTACTTAGGAG-3′). The OsSpo11-5 specific primers for 3′RACE were WP1 (5′-ACGACATTCACTTGGTGGACC-3′) and WP2 (5′-TCTTGACTTGGATG GGCTCC-3′); and for 5′RACE were WP6 (5′-AGCCCATCCAAGTCAAGA-3′) and WP7 (5′-GGTCCACCAAGTGAATGTCGT-3′). The final full-length cDNAs were gel-purified and ligated into the pGEM-T vector (Promega) and confirmed by sequencing. The full-length cDNA sequences of OsSpo11-4 and OsSpo11-5 have been deposited in GenBank, and their NCBI accession numbers are GU177866 and GU170364, respectively.

### Phylogenetic analysis

Phylogenetic analyses were performed to infer the evolutionary relationships of the Spo11, TopVIA, and TopVIB homologues. Homologues of Spo11, TopVIA, and TopVIB in archaea and eukaryotes were identified from public databases using Blast searches. These protein sequences were used as queries for BlastP searches of the NCBI nonredundant database, and TBlastN searches of the NCBI databases of expressed sequence tags (ESTs) and high-throughput genome sequences. Homology of these proteins was conformed by multiple sequence alignments using ClustalX (version 1.8) and phylogenetic analyses using Phyml software (version 3.0, only for Spo11/TopVIA).

### Yeast two-hybrid assay

The MATCHMAKER GAL4 Two-hybrid System 3 (Clontech) was used to detect possible interactions between proteins. The full open reading frame (ORF) sequences of OsSPO11s and OsTOPVIB were amplified by RT-PCR. An amount of 5 g of total RNA extracted from rice flowers was used to synthesize first-strand cDNA with use of SuperScript™ Reverse Transcriptase (Invitrogen) according to the manufacturer's protocols. Primers used for PCR were P1 (5′-ATTGAATTCATGTTGAAAAAAGATCCAAA-3′, *Eco*RI) and P2 (5′-ATCCCGGGCTCGAATCCCAAATCCCC-3′, *Xho*I) for OsSpo11-5, P3 (5′-CACCCGGGTATGGCGGGGAGGGAGAAGAG-3′, *Sma*I) and P4 (5′-TAGCTCGAGGTGCCATACATGTGACTACAG-3′, *Xho*I) for OsSpo11-1, P5 (5′-CGGAATTCGATTCAACGGATGACGATTCG-3′, *Eco*RI) and P6 (5′-ATACTCGAGAGCGAGTCCTCACTGGCTTCAGT-3′, *Xho*I) for OsSpo11-4, and P7 (5′-ATCCCGGGTCGAATCCCAAATCCCCGA-3′, *Sma*I), and P8 (5′-AGGTCGACGACTGCTGAATCGGCAAA-3′, *Sal*I) for OsTOPVIB. The coding regions of the N-terminal 412 amino acids (OsSpo11-5N) and the C-terminal 302 amino acids (OsSpo11-5C, the TopVIA domain) of OsSpo11-5 were amplified with primers P1 and P9 (5′-GTCCTCGAGTCTGAACTGTCACGGTCCAT-3′, *Xho*I), and P10 (5′-ATGAATTCTACTCAGACCAGGATATCCT-3′, *Eco*RI) and P2, respectively. These amplified products were cloned into the AD fusion vector pGADT7 and BD fusion vector pGBKT7, respectively, to construct the OsTopVI-pGADT7 constructs and the OsTopVI-pGBKT7 constructs. These constructs were conformed by sequencing and Western Blot using antibodies against tags of the vectors (HA and Myc, respectively) as described previously (Figure S3) [51]. All the constructs and empty vectors did not show self-activation in yeast. Indicated dual combinations of plasmids were co-transformed into yeast strain AH109 for two-hybrid analysis. Protein–protein interaction was determined by the colony-lift filter assay with X-Gal staining following the Clontech protocol.

### Expression and purification of proteins

The ORF sequences of the three OsSpo11s and OsTOPVIB were amplified by RT-PCR as described previously. The primer pairs used in PCRs were P11 (5′-ATGCTAGCATGTTGAAAAAAGATCCAAA-3′, *Nhe*I) and P12 (5′-AATAGATCTCGCCCTTACTTTGCTGCCA-3′, *Bgl*II) for OsSpo11-5, P13 (5′-CATGCTAGCATGGCGGGGAGGGAGAAGAG-3′, *Nhe*I) and P14 (5′-TAGAGATCTGTGCCATACATGTGACTACAGT-3′, *Bgl*II) for OsSpo11-1, P15 (5′-CTAGCTAGCATGGATGATTCAACGGATGACGA-3′, *Nhe*I) and P16 (5′-ATAAGATCTAGCAGTCCTCACTGGCTTCAGT-3′, *Bgl*II) for OsSpo11-4, and P17 (5′-ATGGATCCCGAATCCCAAATCCCCGA-3′, *Bam*HI) and P18 (5′-GAGAGATCTGACTGCTGAATCGGCAAA-3′, *Bgl*II) for OsTOPVIB. PCR products were gel purified and were digested with *Nhe*I and *Bgl*II (for OsSpo11-1, OsSpo11-4 and OsSpo11-5) or with *Bam*HI and *Bgl*II (for OsTOPVIB). The respective enzyme-digested cDNAs were ligated in-frame into the glutathione S-transferase (GST) gene fusion vector pESP-2, which has an *nmt1* promoter from *Schizosaccharomyces pombe* and a LEU2 selection marker [52], and then confirmed by sequencing. Plasmids of correct construction were transformed into the *S. pombe* strain SP-Q01 competent cells as described previously [52].

Yeast SP-Q01 cells harboring recombinant plasmids from EMM (Q-bio gene) plates were inoculated in EMM liquid media at 30°C. After reaching a mid-log phase (OD600 = 0.5), cells were harvested by centrifugation at 1200×g and washed once with an extraction buffer (0.2 M Tris-HCl, pH 8.0, 10 mM EDTA, 150 mM ammonium sulfate, 50% glycerol, 1 mM PMSF, 2 mM DTT). The pelleted cells were resuspended in 500 ml PBS buffer (0.14 M NaCl, 2.7 mM KCl, 10.1 mM Na_2_HPO_4_, 1.8 mM KH_2_PO_4_, pH 7.3), and then broken by vortexing at 4°C for 5 min in the presence of acid-washed glass beads (425–600 mm in diameter, Sigma). Supernatant was collected by centrifugation at 9500×g. The GST fusion proteins were purified by GST affinity chromatography with Glutathione Sepharose 4B (Amersham) according to the manufacturer's instructions. Finally, the purified native proteins were obtained by removing GST tags with use of biotinylated thrombin (Novagen) (2.5 units/25 µg GST fusion proteins) following the manufacturer's method.

### GST pull-down assay

To generate a polyclonal antibody against OsSpo11-4, a 360-bp fragment of OsSpo11-4 cDNA corresponding to amino acids 1 to 120, which has no similarity to the other 2 OsSpo11 proteins, was amplified with primers P5 and P19 (5′-ATACTCGAGGAGAAACCTTGACTTCCT-3′, *Xho*I), cloned into the *Eco*RI/*Sal*I sites of pET-32a(+) (Novagen), and confirmed by sequencing. *Escherichia coli* BL21 (DE3) cells harboring the recombinant plasmid were cultured in LB medium at 37°C to reach an exponential phase, and thereafter the culture was induced immediately with 0.2 mM IPTG. The recombinant protein purified by Ni^2+^ affinity chromatography (QIAGEN) was used to generate a polyclonal mice antibody against pET-OsSpo11-4.

The GST pull-down assay was performed as described [53], [54]. Briefly, for pull-down assay with purified OsSpo11-4 protein, the full-length OsSpo11-4 ORF sequence amplified with primers P5 and P6 given above was cloned into the *Eco*RI/*Xho*I sites of pET-32a(+) and confirmed by sequencing. pET-OsSpo11-4 fusion protein and pET tag were purified from *E. coli* BL21 (DE3) cells harboring the recombinant plasmids with Ni^2+^ affinity chromatography as described above. pET-OsSpo11-4 and pET tag were changed into 0.5% NP-40 lysis buffer [20 mM Tris-HCl, pH 8.0, 200 mM NaCl, 1 mM EDTA, 0.5% NP-40, 1×cocktail (Roche)], and then were precleared by incubating with Glutathione Sepharose resin preabsorbed by excessive GST proteins on a rocker at 4°C for 2 h. The supernatants were collected and incubated with an equivalent amount of GST-OsTopVIB preabsorbed to Glutathione Sepharose resin on a rocker at 4°C for 6 h. The resulting resins were washed 3 times with the lysis buffer, and thereafter boiled in SDS-PAGE sample buffer to elute bound proteins (termed as pulldowns). The purified pET tag and pET-OsSpo11-4 fusion, the precleared pET tag and pET-OsSpo11-4 fusion, and the GST pulldowns were separated on SDS-PAGE and hybridized with an antibody against pET-OsSpo11-4 fusion protein. SDS-PAGE and western blot were performed as described previously [51].

For pull-down assay using protein extracts, purified yeast-expressed GST-TopVIB or GST (control) as described previously were bound to Glutathione Sepharose 4B resin. Proteins extracted from young rice flowers at the meiosis stage as described [55] were incubated with GST-OsTopVIB or GST preabsorbed to Glutathione Sepharose 4B resin on a rocker at 4°C for 6 h. The resulting Glutathione Sepharose resins were washed 3 times with 0.5% NP-40 lysis buffer and then boiled in SDS-PAGE sample buffer to elute bound proteins. GST and GST-OsTopVIB pulldowns were separated on SDS-PAGE and hybridized with monoclonal antibody against GST and antibody against OsSpo11-4, respectively.

### DNA topoisomerase activity assay

The decatenase activity was examined by use of the Topoisomerase II Assay Kit (Topogen) in a standard reaction mixture (20 µl) containing 2 µl buffer A (50 mM Tris-HCl, pH 8.0, 120 mM KCl, 7.5 mM MgCl_2_, 0.5 mM DTT, and 30 µg/ml BSA), 2 µl buffer B (10 mM ATP), 100 nM of proteins and 0.2 µg of kinetoplast DNA (kDNA). The mixture was incubated at 32°C for 30 min, and then subjected to 1% agarose gel in 1×TAE buffer with ethidium bromide included in the gel. For each assay, 3 independent biological replicates were performed.

The dsDNA cleavage activity was analyzed as described above, except pUC18 plasmid (TIANGEN) was used instead of kDNA as a reaction substrate.

The effects of Mg^2+^ concentration on TopVI activity were determined using standard conditions with changing variable concentrations of Mg^2+^ at a time. DNA bands were visualized by UV, photographed, and analyzed by image Pro-plus software (version 5.1). DNA cleavage reaction rate was determined as a percentage of linear kDNA generated compared to the total kDNA added.

### Semi-quantitative RT-PCR and *in situ* hybridization

The transcript levels of OsSpo11-4 mRNA in different wild-type rice tissues and in RNAi transgenic plants were examined by semi-quantitative RT-PCR with use of SuperScript II RNase^H−^ Reverse Transcriptase (Invitrogen) and primers P5 and P6 given above. The transcript of *tubA* gene (accession No. X91806) served as an internal control [50]. PCR was performed for 26 cycles.

To determine the expression profiles of *OsSpo11-4* in flowers by *in situ* hybridization, flowers were fixed and further processed as described previously [50]. A 360-bp cDNA fragment spanning nucleotides 350 to 710 of OsSpo11-4 ORF, which showed no similarity to the other *OsSPO11* genes, was amplified by use of primers P20 (5′-CCCTGAACTTAACTTGCC-3′) and P21 (5′-AGATAATCCACCTTGACC-3′), cloned into pGEM-T vector (Promega) and confirmed by sequencing. DIG-labeled sense and antisense RNAs were synthesized by *in vitro* transcription (Roche) of the linearized recombinant plasmid. Hybridization was performed as described [50].

### RNA interference and plant transformation

OsSpo11-4 RNAi vector (pOsSpo11-4i) was constructed by inserting a 840-bp fragment specific to OsSpo11-4 cDNA into binary RNAi tool vector pWTC605 derived from pCAMBIA1300 as described [51]. The cDNA fragment was amplified first with the primer pairs P22 (5′-CGTCTAGATGGGAACTGCCAGAGGAGAA-3′, *Xba*I) and P23 (5′-ATGGTACCACCCAACAAATAGATGGCACG-3′, *Kpn*I), or P24 (5′-ATGGTAACCGCCAGAGGAGAAGGTCCAA-3′, *Bst*EII) and P25 (5′-CTGTCGACACCCAACAATAGATGGCACG-3′, *Sal*I). Finally, the 2 digested fragments were ligated into pWTC605 in the sense and antisense orientations, respectively. pOsSpo11-4i was introduced into *Agrobacterium tumefaciens* EH105 and transformed into rice calli to generate OsSpo11-4i plants as described [48]. Seeds of the T0 generation were selected on 1/2 MS medium with 25 mg/l hygromycin B (Roche), and the surviving seedlings were transferred to soil as the T1 generation.

### Southern blot analysis of transgene in RNAi lines

An amount of 20 µg of genomic DNA from RNAi lines was digested completely with *Eco*RV, which has no cut site in the inserted hygromycin phosphotransferase gene sequence. The digested DNAs were separated by electrophoresis on a 0.8% agarose gel and transferred onto a Hybond N^+^ nylon membrane (Amersham). Hybridization was performed at 65°C overnight with the α-^32^P dCTP-labeled 515-bp fragment of the hygromycin phosphotransferase gene. The probe was labeled by use of a primer-a-gene labeling system (Invitrogen) following the manufacturer's instructions.

### Real-time PCR expression analysis

The real-time PCR analysis was performed using primers specific to OsSPO11-4, P26 (GCTTATGATCGTCAGGTTTCTTCAA), and P27 (GGGCCGGGACCTCTGATATA). The expression level of OsSPO11-43 in different RNA samples was calculated according to the internal standard gene, *tubA*, to normalize for variance in the quality of RNA and the amount of input cDNA. The relative expression of OsSPO11-4 in RNAi lines versus wild type was computed by the ΔΔC_T_ method (Applied Biosystems, USA).

### Cytological analysis

For viability assays of mature pollen grains, flowers were randomly collected from RNAi and wild-type plants at the heading stage. Anthers of the sampled flowers were dissected, and pollen grains were stained in Alexander solution [37] and viewed on light microscopy. Meiotic chromosomes were spread and then stained with 4′, 6-diamidino-2-phenylindole (DAPI) following the methods described previously [56], [57].

## Supporting Information

Figure S1 **Schematic genomic structure of** ***OsSPO11-1*** **,** ***4*** **,** ***5*** **and** ***OsTOPVIB*** **genes.** Exons are represented by black boxes. Start and stop codons are shown as arrows above the schematic sequence.
(TIF)

Figure S2 **Multiple alignment of amino acid sequences of OsTopVIB and its homologues using ClustalX software (version 1.8).** Gaps are shown by dashes. Black boxes indicate conserved residues, and grey boxes indicate similar residues. The respective amino acid position of each sequence is given on the right. These sequences are OsTopVIB (AY371050) from *O. sativa*; MtTopVIB (NP_276142) from *Methanobacterium thermoautotrophicum*; AfTopVIB (NP_069486) from *Archaeoglobus fulgidus*; SsTopVIB (O05207) from *Sulfolobus shibatae* and AtTopVIB (AJ297843) from *A. thaliana*.
(TIF)

Figure S3 **Western blot of yeast two hybrid proteins of OsTopVI.** A, Western blot of expressed fusion proteins of the OsTopVI-pGADT7 constructs in yeast strain AH109 using anti-HA antibodies, con refers to protein expressed by the empty pGADT7 vector. B, Western blot of expressed fusion proteins of the OsTopVI-pGBKT7 constructs in AH109 using anti-myc antibodies, con refers to protein expressed by the empty pGBKT7 vector. s5, s5N, s5C, s1, s4 and VIB represent respective HA/myc fusion proteins. M: protein molecular weight.
(TIF)
