# Supplementary figures and images for: OsSpo11-4, a Rice Homologue of the Archaeal TopVIA Protein, Mediates Double-Strand DNA Cleavage and Interacts with OsTopVIB
Source: PLoS One. 2011 May 26;6(5):e20327. doi: 10.1371/journal.pone.0020327 (PMC3102714; doi:10.1371/journal.pone.0020327)

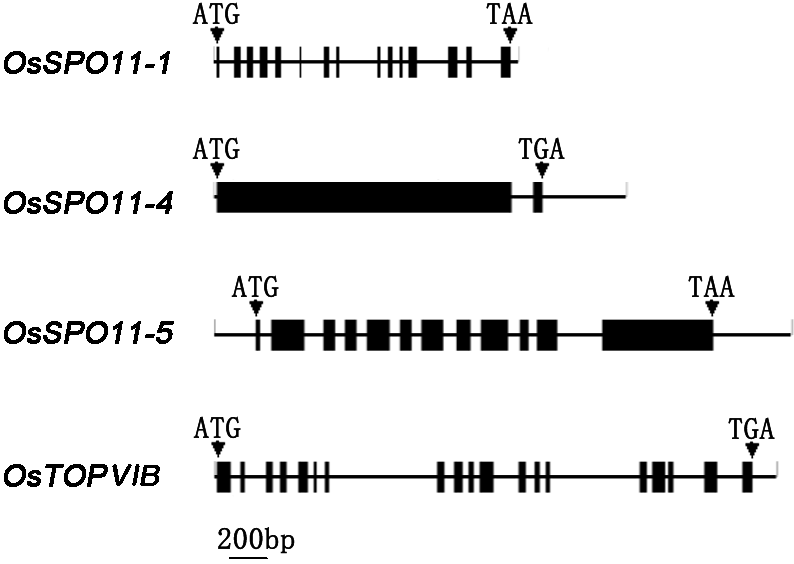

Supplement: Figure S1 — Schematic genomic structure of OsSPO11-1 , 4 , 5 and OsTOPVIB genes. Exons are represented by black boxes. Start and stop codons are shown as arrows above the schematic sequence. (TIF) [file pone.0020327.s001.tif]

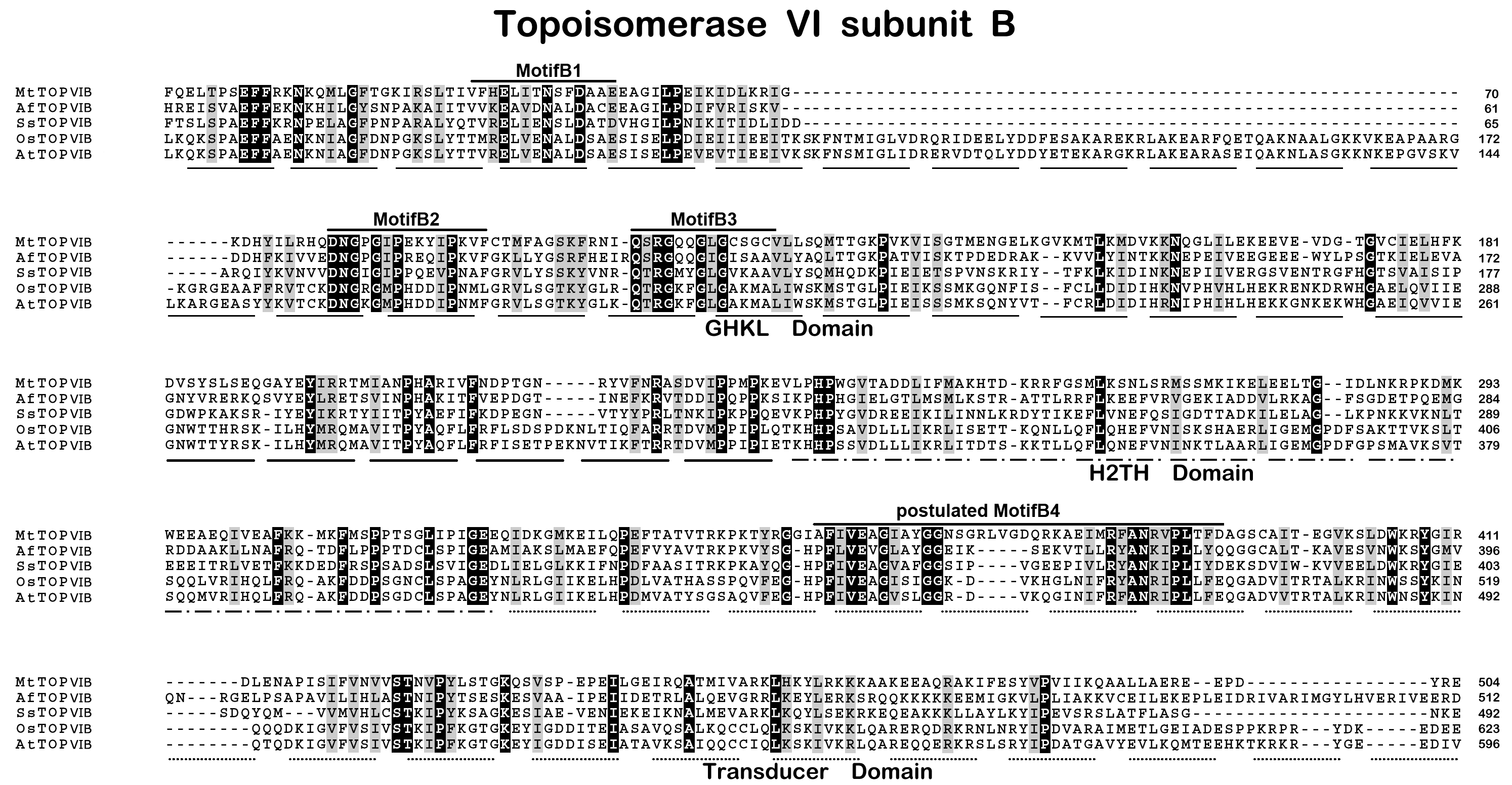

Supplement: Figure S2 — Multiple alignment of amino acid sequences of OsTopVIB and its homologues using ClustalX software (version 1.8). Gaps are shown by dashes. Black boxes indicate conserved residues, and grey boxes indicate similar residues. The respective amino acid position of each sequence is given on the right. These sequences are OsTopVIB (AY371050) from O. sativa; MtTopVIB (NP_276142) from Methanobacterium thermoautotrophicum; AfTopVIB (NP_069486) from Archaeoglobus fulgidus; SsTopVIB (O05207) from Sulfolobus shibatae and AtTopVIB (AJ297843) from A. thaliana. (TIF) [file pone.0020327.s002.tif]

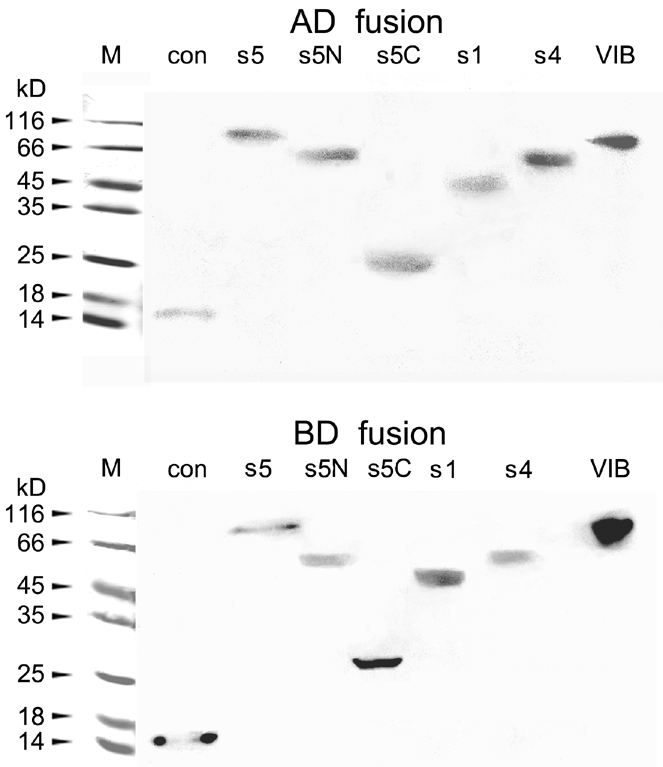

Supplement: Figure S3 — Western blot of yeast two hybrid proteins of OsTopVI. A, Western blot of expressed fusion proteins of the OsTopVI-pGADT7 constructs in yeast strain AH109 using anti-HA antibodies, con refers to protein expressed by the empty pGADT7 vector. B, Western blot of expressed fusion proteins of the OsTopVI-pGBKT7 constructs in AH109 using anti-myc antibodies, con refers to protein expressed by the empty pGBKT7 vector. s5, s5N, s5C, s1, s4 and VIB represent respective HA/myc fusion proteins. M: protein molecular weight. (TIF) [file pone.0020327.s003.tif]
